# BEX1 acts as a tumor suppressor in acute myeloid leukemia

**DOI:** 10.18632/oncotarget.4095

**Published:** 2015-05-26

**Authors:** Oscar Lindblad, Tianfeng Li, Xianwei Su, Jianmin Sun, Nuzhat N. Kabir, Fredrik Levander, Hui Zhao, Gang Lu, Lars Rönnstrand, Julhash U. Kazi

**Affiliations:** ^1^ Division of Translational Cancer Research, Department of Laboratory Medicine, Lund University, Lund, Sweden; ^2^ Lund Stem Cell Center, Department of Laboratory Medicine, Lund University, Lund, Sweden; ^3^ Department of Hematology and Vascular Disorders, Skåne University Hospital, Lund, Sweden; ^4^ School of Biomedical Sciences, Faculty of Medicine, The Chinese University of Hong Kong, Hong Kong; ^5^ Department of Surgery, Faculty of Medicine, The Chinese University of Hong Kong, Hong Kong; ^6^ Laboratory of Computational Biochemistry, KN Biomedical Research Institute, Barisal, Bangladesh; ^7^ Bioinformatics Infrastructure for Life Sciences (BILS), Department of Immunotechnology, Lund University, Lund, Sweden

**Keywords:** FLT3, FLT3-ITD, AML, apoptosis, AKT

## Abstract

Acute myeloid leukemia (AML) is a heterogeneous disease of the myeloid lineage. About 35% of AML patients carry an oncogenic FLT3 mutant making FLT3 an attractive target for treatment of AML. Major problems in the development of FLT3 inhibitors include lack of specificity, poor response and development of a resistant phenotype upon treatment. Further understanding of FLT3 signaling and discovery of novel regulators will therefore help to determine additional pharmacological targets in FLT3-driven AML. In this report, we identified BEX1 as a novel regulator of oncogenic FLT3-ITD-driven AML. We showed that BEX1 expression was down-regulated in a group of AML patients carrying FLT3-ITD. Loss of BEX1 expression resulted in poor overall survival (hazard ratio, HR = 2.242, *p* = 0.0011). Overexpression of BEX1 in mouse pro-B and myeloid cells resulted in decreased FLT3-ITD-dependent cell proliferation, colony and tumor formation, and in increased apoptosis *in vitro and in vivo*. BEX1 localized to the cytosolic compartment of cells and significantly decreased FLT3-ITD-induced AKT phosphorylation without affecting ERK1/2 or STAT5 phosphorylation. Our data suggest that the loss of BEX1 expression in FLT3-ITD driven AML potentiates oncogenic signaling and leads to decreased overall survival of the patients.

## INTRODUCTION

FMS-like tyrosine kinase-3 (FLT3) is a receptor tyrosine kinase belonging to the type III receptor tyrosine kinases family. FLT3 expression has been detected in almost all acute myeloid leukemia (AML) patients, and the activating mutations in FLT3 occur in as high as 35% of AML patients [1] and less frequently in acute lymphoblastic leukemia (ALL) patients [2]. The most common FLT3 mutation is an internal tandem duplication (ITD), and other oncogenic mutations include point mutations in the kinase domain. Clinically, FLT3-ITD mutations are seen frequently in AML with normal karyotype, t(6:9), t(15:17) and trisomy 8 [3, 4] where it significantly increases the risk of relapse without affecting complete remission rates [5]. Consequently, FLT3-ITD expression limits disease-free and overall survival [6]. FLT3-ITD is an in frame duplication of 3 to 400 base pairs occurring in the region of the gene encoding the juxtamembrane domain of the receptor, and the length of the ITD mutation correlates with overall survival [7]. Thus, FLT3 is an attractive target to inhibit in AML patients with constitutive active FLT3 mutants. Wild-type FLT3 and its oncogenic mutants activate several downstream signaling cascades including PI3K-AKT and MAPK pathways resulting in cell survival [8–14]. Additionally FLT3-ITD activates STAT5 signaling [15].

BEX1 belongs to the Brain-Expressed X-linked (BEX) gene family. The initial description of the BEX genes was made in 1999 and included three mouse genes on the X chromosome with high expression levels in the brain. Up to six paralog BEX genes have since been identified in rodents and humans. BEX1 and its closest homolog BEX2 share a similar protein sequence (87% identity), while BEX3/NADE, which was characterized as an interactor of the neurotrophin receptor p75NTR death domain, is only 30% identical to either BEX1 or BEX2 and represents a more distant member of the family [16–21]. Human BEX1 is located at Xq22.1 while BEX2 is located at Xq22.2. Both BEX1 and BEX2 contain a characteristic BEX domain. Human BEX1 is expressed in the central nervous system with high levels in pituitary, cerebellum, and temporal lobe, but also widely expressed outside of the central nervous system with high expression in the liver [19, 22]. A later study showed that BEX1 interacts with p75NTR regulating the cell cycle and neuronal differentiation in response to nerve growth factor (NGF) [23]. BEX1 and BEX2 have also been shown to act as a tumor suppressor in malignant glioma [24]. Furthermore, the higher BEX1 expression was detected in AML cell lines with MLL-mutations compared to MLL-WT cell lines [25]. Treatment with the hypomethylating agent 5-Aza and with the HDAC inhibitor TSA induced expression of BEX1 in MLL-WT cells indicating that BEX1 is epigenetically regulated [25–27]. In the BCR-ABL positive K562 cell line, silencing of BEX1 in association with protocadherin 10 (PSDH10) induced resistance to imatinib [28]. In conclusion, current studies suggest that BEX1 is expressed in a variety of cells where it acts as a tumor suppressor.

In this report, we show that BEX1 is down-regulated in a group of FLT3-ITD driven AML patients. Loss of BEX1 expression resulted in activation of oncogenic signaling and reduced patient overall survival. BEX1 localized to the cytosolic compartments and overexpression of BEX1 resulted in decreased cell proliferation and colony formation, delayed tumor formation and increased apoptosis by inhibiting AKT signaling induced by FLT3-ITD.

## RESULTS

### BEX1 expression is downregulated in the MV4-11 compared to the MOLM-13 cell line

**Figure 1:**
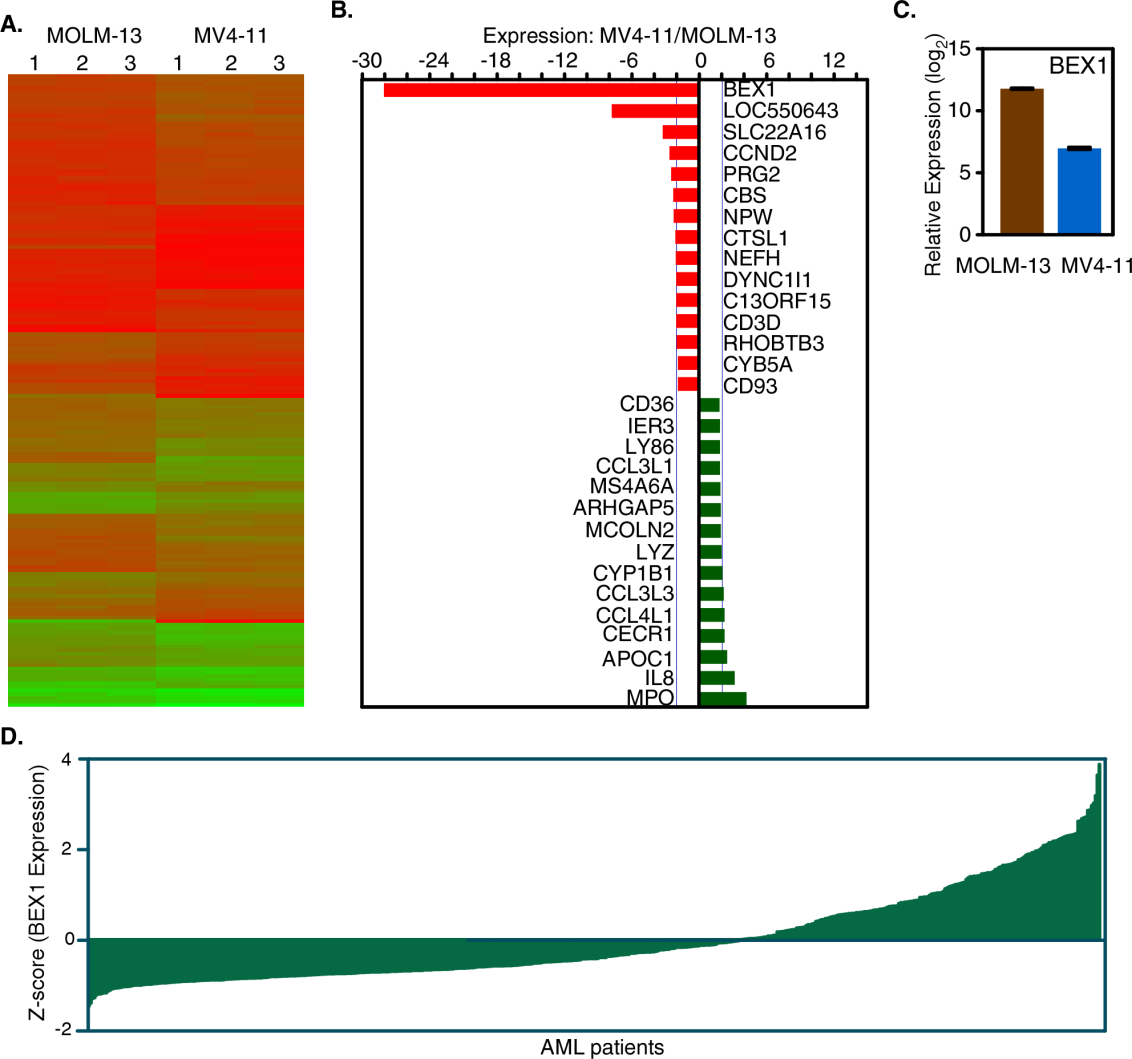
Deregulated gene expression in MV4-11 and MOLM-13 cell lines **A.** Deregulated gene expression patterns in MV4-11 and MOLM-13 cell lines. **B.** Up-regulated and down-regulated genes in MV4-11 versus MOLM-13 cell lines. **C.** Relative BEX1 expression in MOLM-13 and MV4-11 cell lines. **D.** The BEX1 expression is deregulated in AML patients. Data set GSE14468 was used.

In our previous study, we observed that MV4-11 and MOLM-13 cell lines displayed differential response to drug-induced apoptosis, where MOLM-13 cells were more sensitive than MV4-11 [29]. To understand the basic differences between those cell lines we analyzed gene expression using microarray. We observed that both cell lines displayed differences in gene expression patterns (Fig. 1A) suggesting that although both cell lines are known to be dependent on FLT3-ITD, additional genetic and epigenetic mutations in different genes led to expression of unique genes in each cell lines. Genes up-regulated or down-regulated in the respective cell lines were determined by SAM tools. We observed that several genes displayed significant up-regulation or down-regulation. *BEX1, LOC550643, SLC22A16, CCND2, PRG2, CBS and NPW* genes were down-regulated in MV4-11 cells, whereas *MPO, IL8, APOC1, CECR1 and CCL4L1* genes were up-regulated (Fig. 1B). Interestingly *BEX1* expression was found to be 28-fold down-regulated in MV4-11 cells (Fig. 1C) compared to MOLM-13 cells. However, next-generation sequencing of MOLM-13 and MV4-11 cell lines did not identify any loss-of-function mutations in BEX1 gene in the MV4-11 cell line (data not shown). Furthermore, we observed differential BEX1 expression in a data set of primary AML patient samples (Fig. 1D). Therefore, we suggest that BEX1 expression is down-regulated in a group of AML patients.

### Loss of BEX1 expression correlates with poor survival of FLT3-ITD positive AML patients

**Figure 2:**
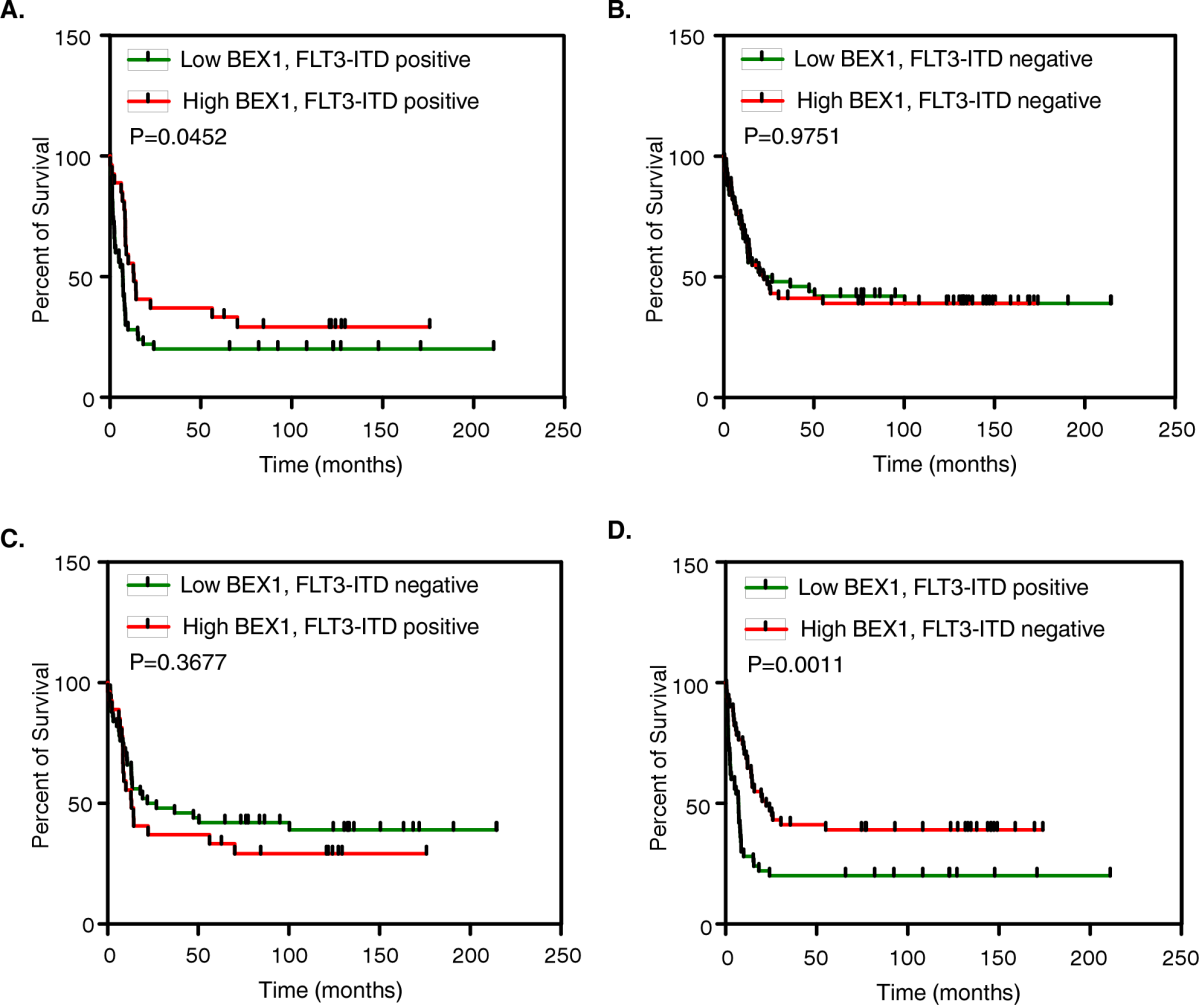
Overall survival of AML patients with higher and lower BEX1 expression Data set GSE14468 was used in this analysis. Z-score was used to divide higher (*n* = 50) and lower (*n* = 50) BEX1 expressing patients. **A–D.** Overall survival of AML patients with FLT3-ITD positive and BEX1 higher or lower expression (A), FLT3-ITD negative and BEX1 higher or lower expression (B), FLT3-ITD positive plus BEX1 higher versus FLT3-ITD negative plus BEX1 lower expression (C) and, FLT3-ITD negative plus BEX1 higher versus FLT3-ITD positive plus BEX1 lower expression (D).

Because we observed that *BEX1* was down-regulated in MV4-11 cells and a group of AML patients, we hypothesized that *BEX1* may play a role in AML. We analyzed the prognostic significance of *BEX1* in AML using gene expression data (GSE6891, *N* = 525) of primary AML patient samples. We observed that the loss of BEX1 expression significantly correlated with poor overall survival in patients carrying FLT3-ITD and reduced median survival of around 50% (HR 1.697, *p* = 0.0452) (Fig. 2A). Furthermore, comparison between FLT3-ITD negative patients and BEX1 higher and lower expression (Fig. 2B), and patients with lower BEX1 expression and FLT3-ITD negative versus higher BEX1 and FLT3-ITD positive (Fig. 2C) did not display any difference in patient survival. The patient group with lower BEX1 and FLT3-ITD mutation versus higher BEX1 expression without FLT3-ITD mutation displayed a significant difference in patient survival (HR 2.242, *p* = 0.0011) (Fig. 2D). With other deregulated genes, we did not observe any significant correlation (Fig. S1A–S1D). The BEX1 expression did not display any correlation to the overall survival of the entire patient group regardless of FLT3-ITD mutation (Fig. S1E). Therefore, we suggest that the loss of BEX1 expression in AML patients carrying an FLT3-ITD mutation leads to an elevated risk compared to other groups of patients.

### Loss of BEX1 expression correlates with up-regulation of survival pathways

**Figure 3:**
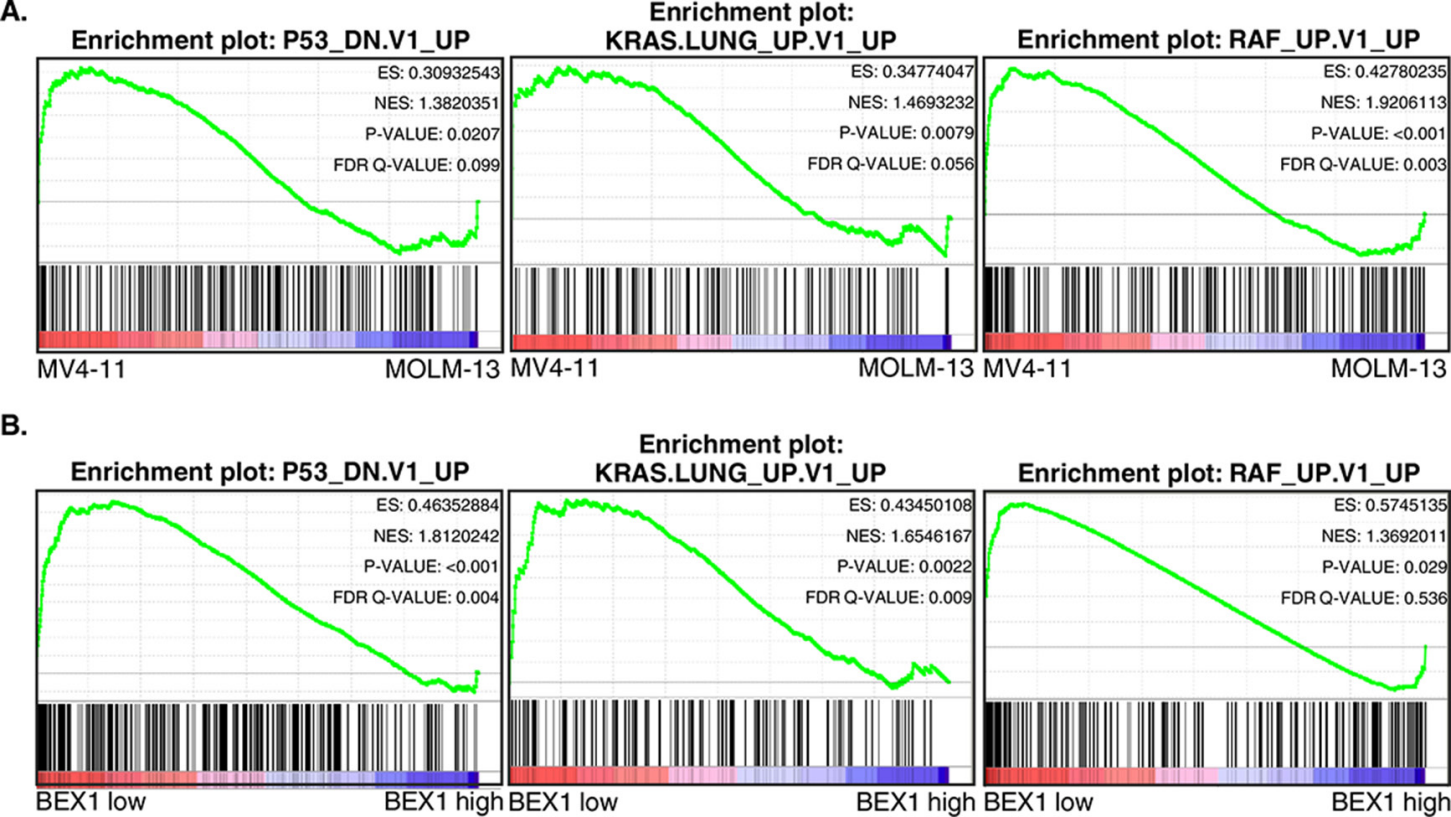
GSEA showed enrichment of oncogenic pathways in lower BEX1 expressing cells and patients Data set GSE14468 was used in this analysis. Z-score was used to divide higher (*n* = 50) and lower (*n* = 50) BEX1 expressing patients. **A.** MV4-11 cells display enrichment of several oncogenic pathways in comparison to MOLM-13 cells. **B.** AML patients with lower BEX1 expression showed enrichment of several oncogenic pathways compared to patients with higher BEX1 expression.

Since the loss of BEX1 expression correlated with poor survival in FLT3-ITD positive patients, we wanted to analyze whether loss of BEX1 expression results in up-regulation of any oncogenic pathways. To that end, we analyzed enrichment of oncogenic pathways using gene set enrichment analysis (GSEA). We observed enrichment of several oncogenic pathways including loss of p53 function, KRAS and RAF pathways in MV4-11 cells in comparison with MOLM-13 cells (Fig. 3A). Moreover, similar enrichment of pathways was observed in FLT3-ITD positive AML patients with lower BEX1 expression (Fig. 3B). These results indicate a possible link between the loss of BEX1 expression and enhancement of oncogenic signaling in AML, which has already been shown in other malignancies [24].

### BEX1 expression leads to impaired cell proliferation, inhibits colony formation and induces apoptosis

**Figure 4:**
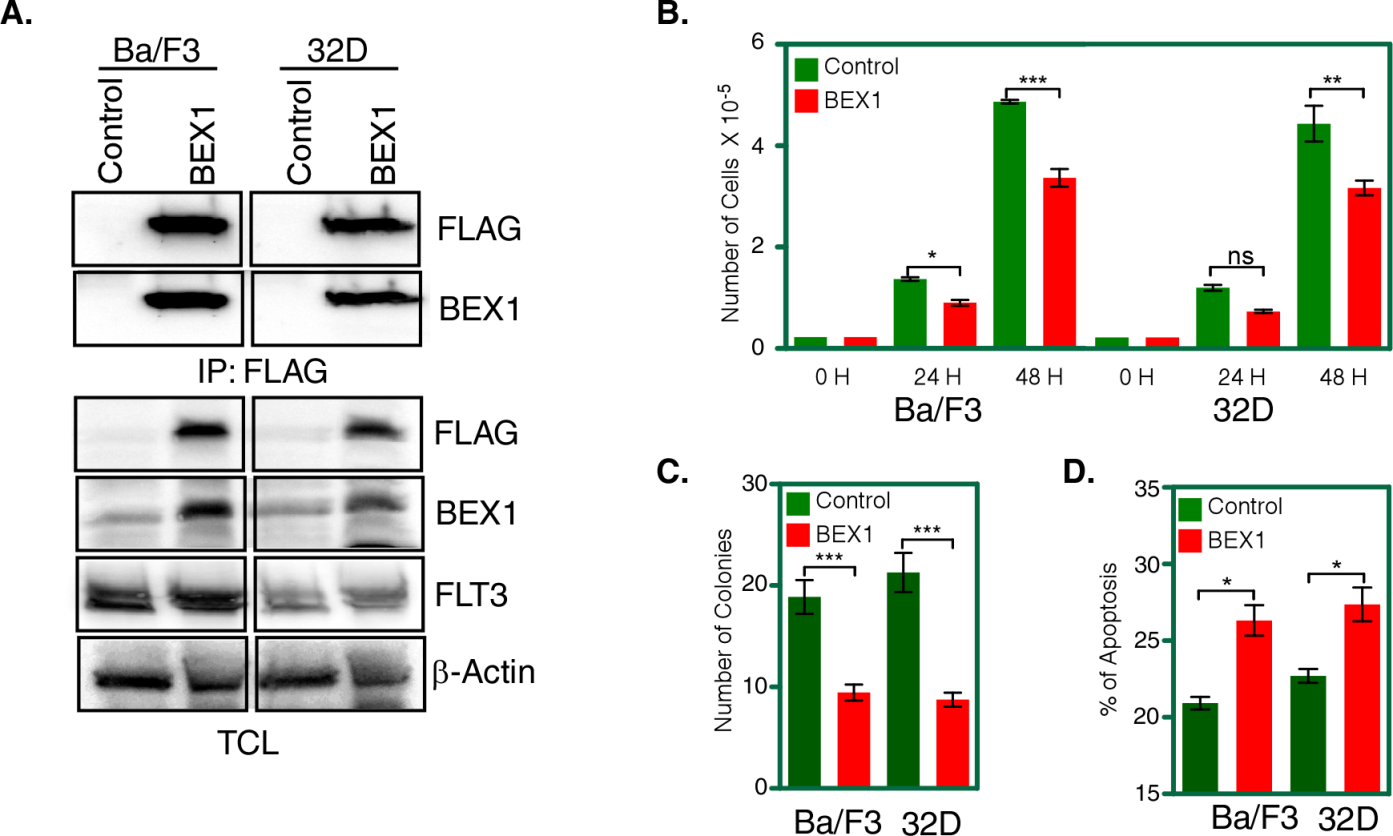
BEX1 expression inhibited cell proliferation, colony formation and enhanced apoptosis Cells were washed three times with RPMI-1640 to remove IL3. **A.** Expression of BEX1 and FLT3-ITD in stably transfected Ba/F3 and 32D cells was measured by western blotting analysis. **B.** FLT3-ITD dependent cell proliferation in presence and absence of BEX1 expression was measured after 24 and 48 hours using stably transfected Ba/F3 and 32D cells. **C.** Stably transfected Ba/F3 and 32D cells were used to determine colony formation potential in the semi-solid medium. **D.** Apoptosis induced by BEX1 expression was measured using Annexin-V and 7-AAD kit.

Results from survival assays and GSEA suggest that BEX1 plays a role in FLT3-ITD positive AML patients. To assess the role of BEX1 in FLT3-ITD signaling we generated two cell lines by stably transfecting FLT3-ITD along with BEX1 or empty control vector in the pro-B cell line Ba/F3 and the myeloid cell line 32D. Expression of FLT3-ITD and BEX1 was verified by western blotting (Fig. 4A). Expression of BEX1 significantly reduced FLT3-ITD-dependent cell proliferation of both Ba/F3 and 32D cells (Fig. 4B). Furthermore, cells expressing BEX1 displayed reduced number of colonies in semi-solid medium (Fig. 4C) and significantly enhanced apoptosis (Fig. 4D) in both cell lines, suggesting that BEX1 expression is essential for controlling FLT3-ITD-induced biological events.

### BEX1 expression leads to delayed tumor formation in a mouse xenograft model

**Figure 5:**
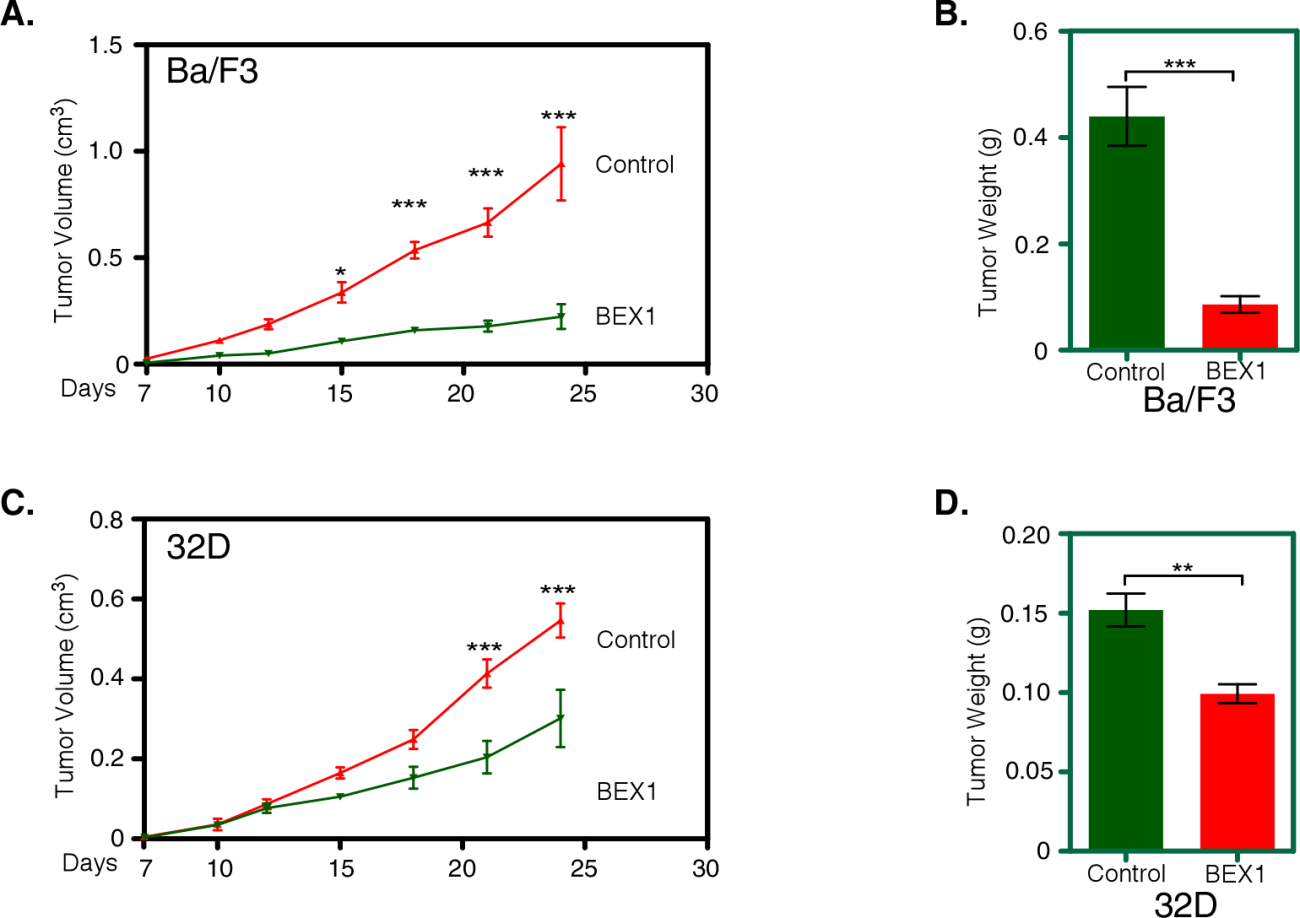
BEX1 delayed tumor growth in mouse xenograft Cells were washed three times with RPMI-1640 to remove IL3. **A–D.** Cells expressing BEX1 or control were xenografted into mice and tumor growth was monitored for 24 days. Tumor volume (A) and weight (B) in Ba/F3 cells as well as in 32D cells (tumor volume (C) and weight (D)) were analyzed.

Because BEX1 expression reduced cell proliferation, inhibited colony formation and induced apoptosis, we aimed to check whether BEX1 expression delays FLT3-ITD-induced tumor formation in mice. Nude mice were injected subcutaneously with Ba/F3-FLT3-ITD and 32D-FLT3-ITD cells along with BEX1 or with empty control vector. We observed that BEX1 expression significantly reduced tumor volume (Fig. 5A) and tumor weight (Fig. 5B) in Ba/F3 as well as in 32D cells (Fig. 5C and 5D) in xenografted mice.

### BEX1 localizes to the cytoplasm but does not affect FLT3 stability

**Figure 6:**
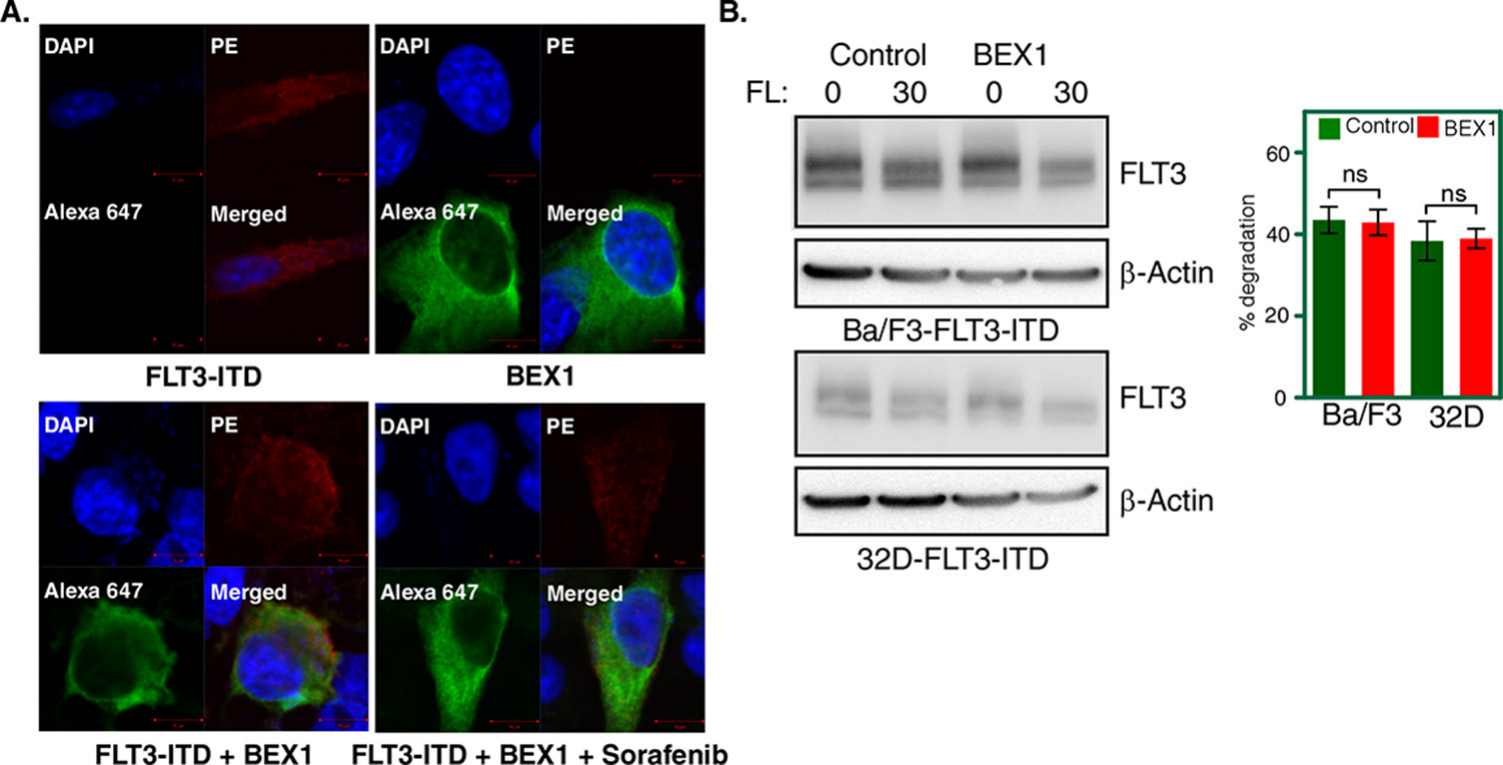
BEX1 localized to the cytosol and did not affect FLT3-ITD stability **A.** Localization of BEX1 was visualized by confocal microscope. FLT3-ITD was stained with PE-conjugated antibody and BEX1-FLAG was stained with Alexa Flour 647-conjugated anti-FLAG antibody. **B.** Cells were washed three times with RPMI-1640 to remove IL3. FLT3 degradation was measured in transfected Ba/F3 and 32D cells after 30 minutes of ligand stimulation.

To understand how BEX1 acts on FLT3-ITD-induced leukemogenesis, we first checked sub-cellular localization of BEX1 in FLT3-ITD expressing cells. We observed that BEX1 localized to the cytosolic compartment of cells and that localization was independent of FLT3-ITD activity (Fig. 6A) suggesting that FLT3-ITD is not the direct target of BEX1. Furthermore, BEX1 expression did not alter ubiquitination or tyrosine-phosphorylation (data not shown) nor did it influence the degradation of FLT3-ITD (Fig. 6B). In addition, we were unable to detect any interaction in between FLT3-ITD and BEX1 (data not shown). Thus, it is more likely that FLT3-ITD is not itself a target of BEX1 but that signaling proteins downstream of FLT3-ITD might be a target.

### BEX1 expression selectively inhibits FLT3-ITD-induced AKT phosphorylation

**Figure 7:**
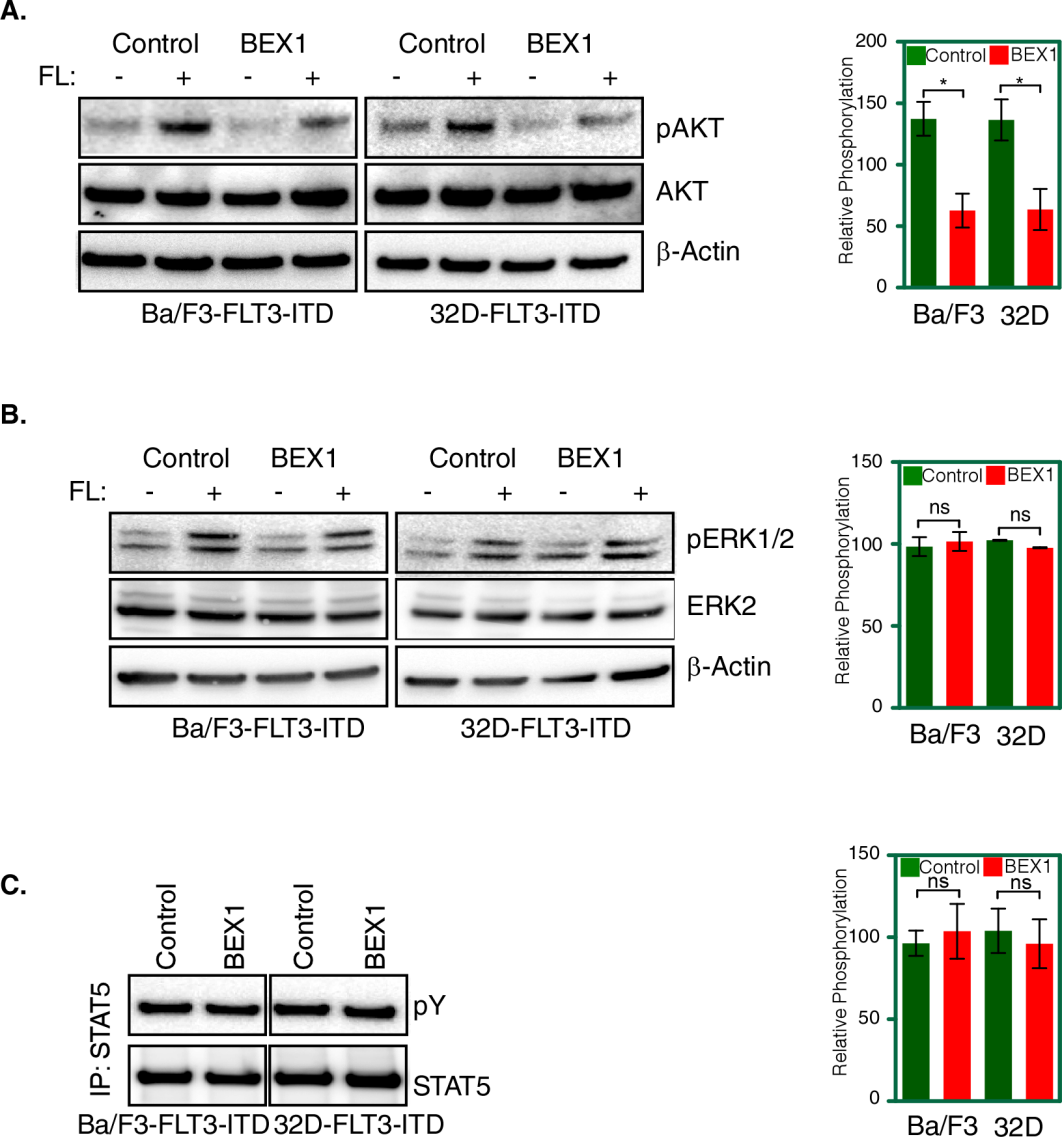
BEX1 expression decreased AKT phosphorylation Cells were washed three times with RPMI-1640 to remove IL3. **A–B.** Transfected Ba/F3 and 32D cells were stimulated with a ligand for 5 minutes before lysis. Total cell lysates were used for SDS-PAGE and western blotting analysis with AKT (A) and ERK (B) antibodies. Ligand stimulated samples were used for quantification. **C.** Cell lysates for stimulated and unstimulated cells were immunoprecipitated with an anti-STAT5 antibody followed by western blotting analysis.

Because BEX1 did not alter FLT3-ITD stability or tyrosine-phosphorylation, we analyzed FLT3 downstream signaling using phospho-specific antibodies. We observed that BEX1 expression significantly blocked AKT phosphorylation (Fig. 7A) in both Ba/F3 and 32D cell lines, but did not block ERK1/2 phosphorylation (Fig. 7B) or STAT5 phosphorylation (Fig. 7C). Thus, we suggest that BEX-1 inhibits FLT3-ITD signaling by blocking FLT3-ITD-induced AKT phosphorylation.

## DISCUSSION

In this study, we aimed to address the role of BEX1 in FLT3-ITD expressing AML. The BEX1 expression was down-regulated in MV4-11 cells and also a subset of AML patients, and loss of BEX1 expression correlated with poor overall survival. BEX1 localized to the cytosol and controlled FLT3-ITD signaling by negative regulation of AKT phosphorylation.

The role of BEX1 in human cancer has not been thoroughly studied. BEX1 has been shown to be overexpressed in ER-positive breast cancer, but its role has not been defined [30]. In this study, we observed that BEX1 expression was down-regulated in MV4-11 cells compared to MOLM-13 cells as well as in a group of AML patients. AML patients positive for FLT3-ITD mutation along with reduced BEX1 expression displayed poor overall survival suggesting that BEX1 acts as a tumor suppressor in AML. A role of BEX1 as a tumor suppressor has previously been suggested in malignant glioma, where BEX1 expression was silenced by extensive promoter hyper-methylation [24]. Next generation sequencing of MV4-11 and MOLM-13 cells did not identify any mutations in the BEX1 gene, suggesting that BEX1 expression was probably also down-regulated due to the epigenetic modifications in MV4-11 cells as well as in a group of AML patients similar to in malignant glioma. It will be of interest to analyze the promoter region of those cell lines as well as of AML patient samples.

A demethylating agent, azacytidine, is being used in the clinic for treating patients with myelodysplastic syndrome (MDS) [31]. It is also used for AML patients to keep the disease under control when conventional cytostatics failed or when the physical status or the age of the patient does not permit intensive therapy. Azacytidine enhances the efficacy of chemotherapy [32] probably through inducing expression of BEX1, as this drug has been shown to induce expression of BEX2 in MLL-positive AML cell line [26, 27]. Expression of BEX1 increased the sensitivity to chemotherapy-induced apoptosis in malignant glioma [24] and, furthermore, down-regulation of BEX1 in the BCR-ABL positive K562 cell line led to resistance to imatinib treatment [28, 33]. Therefore, AML patient with loss of BEX1 expression might benefit from azacytidine treatment combined with conventional cytostatics in order to lower the risk of relapse. In fact, a recently presented phase-2 study showed promising results in treating refractory FLT3-ITD positive AML with a combination of sorafenib and azacytidine [34].

The observation that BEX1 expression promotes apoptosis and inhibits cell proliferation, colony formation and tumor formation induced by FLT3-ITD suggests that BEX1 expression is favorable for AML patients who are positive for the FLT3-ITD mutation. A recent study suggested that BEX1 expression is suppressed in pediatric intracranial ependymoma due to epigenetic modifications and that overexpression of BEX1 significantly suppressed cell proliferation and colony formation in cell lines [35], in line with our observation that BEX1 acts as a tumor suppressor. The mechanism by which BEX1 displays its tumor suppressor activity might be cellular context dependent. It has been shown that BEX1 suppresses NF-κB signaling in oral squamous cell carcinoma [36]. In our study, we observed that BEX1 selectively suppresses AKT phosphorylation without affecting ERK1/2 and STAT5 phosphorylation suggesting that BEX1 controls FLT3-ITD signaling by blocking AKT activation. Although BEX1 inhibited FLT3-ITD-induced AKT phosphorylation, it neither interacted with FLT3-ITD nor regulated FLT3-ITD activation or stability. These observations indicate that FLT3-ITD is not a direct target of BEX1-mediated regulation. Otherwise it would also inhibit ERK1/2 and STAT5 phosphorylation, but a selective regulator of FLT3-ITD-induced AKT signaling.

Taken together, our study suggests that BEX1 has a tumor suppressor role in AML and that loss of BEX1 expression results in poor overall survival in FLT3-ITD positive AML patients. Since BEX1 is capable of limiting cell proliferation, colony formation, tumor formation and inducing apoptosis, drugs that enhance BEX1 expression would be beneficial for the treatment of patients with loss of BEX1 expression in FLT-ITD driven AML.

## MATERIALS AND METHODS

### Cell culture

The human AML cell lines, MV4-11, and MOLM-13, were maintained in RPMI-1640 media (Hyclone, Thermo Scientific, Waltham, MA) supplemented with 10% heat-inactivated fetal bovine serum (Life Technologies, Carlsbad, CA) and 1% penicillin and streptomycin. The murine hematopoietic cell line Ba/F3 and the myeloid cell line 32D were cultured in the same medium with addition of 10 ng/ml murine interleukin 3 (IL3) as recommended before [37]. COS-1 cells were cultivated in DMEM supplemented with 10% heat-inactivated fetal bovine serum (Life Technologies, Carlsbad, CA) and 1% penicillin and streptomycin. Cells were grown at 37°C in a humidified atmosphere containing 5% CO_2_.

### Plasmids, antibodies and inhibitors

Plasmid expressing human BEX1, pCMV- BEX1-WT-Myc-DDK (FLAG) was purchased from Origene, Rockville, MD. For retroviral transduction, the pMSCV-BEX1-WT-Myc-FLAG plasmid was generated by ligating full-length BEX1 into the pMSCVneo vector. Anti-FLT3 antibody was described previously [38]. Anti-phosphotyrosine antibody 4G10 was from Millipore (Life Technologies, Carlsbad, CA). Anti-phospho AKT was from Epitomics (Abcam, Cambridge, UK) and anti-phospho ERK antibody was form Santa-Cruz, Dallas, Texas. Anti-β-actin antibody was from Sigma-Aldrich, St. Louis, MO. Flag-Alexa 647 was from Cell Signaling Technology, Inc. Danvers, MA. DAPI was from Molecular Probes. FLT3-PE was from BD Biosciences Franklin Lakes, New Jersey.

### Stable transfection of Ba/F3 and 32D cells

To establish Ba/F3 and 32D cells stably expressing FLT3-ITD, EcoPack packaging cells were transfected with pMSCV-puro-FLT3-ITD construct, and virus-containing supernatants were collected 72 h after transfection. Retroviral infection of Ba/F3 and 32D cells was followed by a 2-week selection in 1.2 μg/ml puromycin. Expression of FLT3-ITD was confirmed by flow cytometry and western blotting. FLT3-ITD-transfected Ba/F3 and 32D cells were then further transfected with the pMSCV-neo-BEX1-Myc-FLAG construct or empty vector. Cells were selected with 0.8 mg/ml G-418 for 2 weeks, and BEX1 expression was verified by Western blotting.

### Immunoprecipitation and western blotting

After required treatments such as ligand-stimulation, cells were washed once with cold PBS. Cells were then lysed using Triton X-100 based lysis buffer. Cell lysates were mixed with DDT and SDS containing loading buffer in a 1:1 ratio and boiled before separation by SDS-PAGE. For immunoprecipitation cell lysates were mixed with specific primary antibodies for 1 hour on ice followed by purification on protein G Dynabeads and SDS-PAGE analysis.

### Apoptosis

Apoptosis was measured using annexin V and 7-aminoactiomycin D (7-AAD) kit (BD biosciences). Cells positive for annexin V or both annexin V and 7-AAD were counted as apoptotic cells.

### Cell proliferation

Cells were seeded in a 24-well plate and incubated for 48 hours. Living cells were stained with trypan blue at 24 h and 48 h and counted with a Countess cell counter.

### Colony formation assay

Around 500 cells were seeded in semisolid methylcellulose medium (Stem Cell Technologies). Cells were cultured for seven days before counting colonies.

### Exome sequencing

Total genomic DNA was extracted from cell lines using DNeasy Blood and Tissue kits (Qiagen). Human All Exon enrichment (Agilent SureSelectXT) library was used to read 100 bp paired-end sequencing on a Genome Sequencer Illumina HiSeq2500.

### Microarray analysis

Triplicate samples from MV4-11 and MOLM-13 cells were used. Cells were cultured normally using standard growth medium as mentioned above. Total RNA was extracted from cells using RNeasy mini kit (Qiagen). Illumina bead array technology was used to analyze mRNA expression using Illumina HumanHT-12 v4 Expression BeadChip. Gene expression was compared using significance analysis of microarrays (SAM) tools [39] and gene set enrichment analysis (GSEA) [40]. SCIBLU facility at Lund University was used for microarray analysis.

### Confocal microscopy

COS-1 cells were transiently transfected with either pcDNA3-Flt3-ITD, pCMV-BEX1-Myc-FLAG or both using Lipofectamine 2000. Sorafenib were added to some samples (50 nM), and cells were incubated overnight. Cells were then fixed in 4% para-formaldehyde in PBS and incubated for 30 min. Blocking and permeabilization were done by adding a mixture of 0.5% Triton-X100 in PBS and 5% goat serum. Finally cells were stained and washed before examination with confocal microscopy.

### Mouse xenograft

Briefly, 0.1 ml PBS and Matrigel (1:1) containing 2 × 10^6^ control or BEX1 expressing BaF3 or 32D cells were injected subcutaneously into 4-week old male BALB/c nude mice, 5 mice in each group. Animals were monitored for weight change and tumor size. Afterward the mice were maintained for 24 days before the tumors were collected.

### Quantification of western blots and statistical analysis

Western blots were quantified using ImageJ. Target signals were normalized against loading control β-actin. One-way ANOVA was used for statistical analysis. In statistical significance tests, “ns” represents not significant, “*” represents *p* < 0.05, “**” represents *p* < 0.01, and “***” represents *p* < 0.001.

## SUPPLEMENTARY FIGURES


