# Supplementary figures and images for: BEX1 acts as a tumor suppressor in acute myeloid leukemia
Source: Oncotarget. 2015 May 26;6(25):21395–405. doi: 10.18632/oncotarget.4095 (PMC4673273; doi:10.18632/oncotarget.4095)

## SUPPLEMENTARY FIGURES

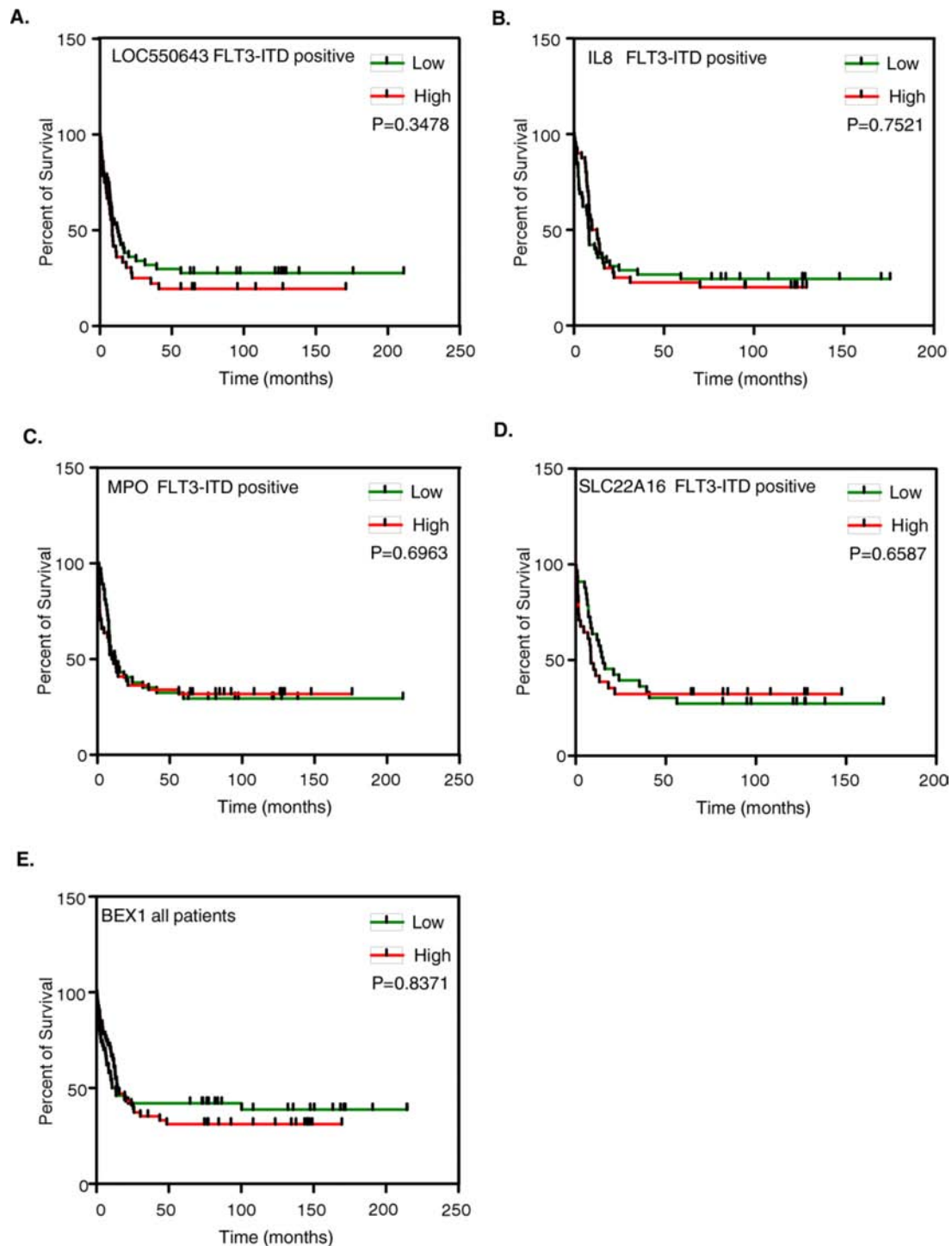

Supplementary Figure S1: Overall survival.

Supplement: Supplementary file 1 [file oncotarget-06-21395-s001.pdf]
